# Diversity of the cladocerans (Crustacea, Branchiopoda) in the Republic of Tyva, Russian Federation

**DOI:** 10.3897/BDJ.13.e163656

**Published:** 2025-10-06

**Authors:** Nadezhda Kirova, Valeria Kirova, Alexey Kotov

**Affiliations:** 1 Tuvinian Institute for the Exploration of Natural Resources of the Siberian Branch of Russian Academy of Sciences, Kyzyl, Russia Tuvinian Institute for the Exploration of Natural Resources of the Siberian Branch of Russian Academy of Sciences Kyzyl Russia; 2 National Research Nuclear University MEPhI, Moscow, Russia National Research Nuclear University MEPhI Moscow Russia; 3 A.N. Severtsov Institute of Ecology and Evolution of the Russian Academy of Sciences, Moscow, Russia A.N. Severtsov Institute of Ecology and Evolution of the Russian Academy of Sciences Moscow Russia

**Keywords:** field studies, occurrence, Palaearctic, species richness, benthos, zooplankton, biodiversity, GIB

## Abstract

**Background:**

The cladoceran fauna is well studied across the Palaearctic, but remains poorly known in the Republic of Tyva, Russia. Our database represents the results of the faunistic survey of the cladocerans in this region performed during almost 30 years (1993–2022). A total of 902 sites were investigated, most of them being located in mountain areas (49°45' – 53°46' North latitude and 88°49' – 98°56' East longitude). The dataset includes the cladocerans sampled from permanent and temporary waterbodies with varying depths, altitudes and salinity levels. Sampling was conducted using plankton nets and then samples were transported to the Tuvinian Institute for Exploration of Natural Resources of the Siberian Branch of RAS for identification and further examined at the A.N. Severtsov Institute of Ecology and Ecology RAS. Species were identified, based on recent monographs and juvenile specimens (sometimes indeterminable, based on morphological methods) were excluded from the dataset. The dataset was published as a Darwin Core Archive in GBIF. For each sampling event, the coordinates of the location, date and collector are recorded.

**New information:**

The dataset contains information on zooplankton and microzoobenthos from numerous permanent and temporary waterbodies in the Republic of Tyva, Russian Federation. Previously, the region’s cladoceran fauna was poorly studied.

Our core data table includes 3,599 records representing 76 species from 902 locations. The most species-rich families are Chydoridae (30 species from 17 genera) and Daphniidae (26 species from 5 genera). No invasive species were detected.

The results of this study contribute to a deeper understanding of the plankton and microzoobenthos communities in the Central Asian mountain regions.

## Introduction

Water fleas (Crustacea, Cladocera) are common in various types of continental waterbodies and often dominate in freshwater zooplankton and microzoobenthos (Dumont and Negrea 2002). Moreover, they serve as model organisms in various branches of biological science (Lampert 2011, Smirnov 2017). However, their study is uneven across different regions of the world, including within the Palaearctic. The northern regions, particularly Europe and the Far East, are relatively well studied (Alonso 1996, Kotov et al. 2011, Korovchinsky et al. 2021a). In contrast, hydrobiologists have paid much less attention to the southern regions of Central Asia, such as the Middle Asian countries, Mongolia and southern Siberia (Alonso 2010, Dadykin et al. 2024).

These regions are home to several major mountain ranges, including the Pamirs, Tien Shan and Sayans. Mountain ecosystems are known for their unique fauna, often containing local endemics. During the Pleistocene glacial cycles, several refugia for microscopic freshwater organisms were located here (Hewitt 2000, Zuykova et al. 2019). Despite this, relatively few studies on Cladocera focus on these areas.

The Republic of Tyva is amongst such underexplored regions. The first records of crustaceans from this region date back to the early 20^th^ century (Sars 1903). During the Soviet era, a number of publications related to fisheries in Tyva appeared (Greze and Greze 1958, Gundrizer 1975).

A systematic study of Tuvinian water fleas began in the 1990s. Since then, several papers have been published (Kotov 1999, Kirova 2021, Sinev et al. 2021, Kirova and Oydup 2022). However, only a small proportion of the region’s waterbodies have been sampled so far.

A detailed study of the cladoceran fauna in Tyva is urgently needed and a formal inventory is the first step in this direction.

## General description

### Purpose

The aim of this paper was to describe the current cladoceran fauna of the Republic of Tyva, based on a recently published dataset, which contains results of the survey in this region performed during almost 30 years (1993–2022) (Kirova et al. 2024).

## Project description

### Title

Diversity of the cladocerans (Crustacea, Branchiopoda) in the Republic of Tyva, Russian Federation

### Personnel

Nadezhda A. Kirova, Valeria O. Kirova, Alexey A. Kotov

### Study area description

**Figure 1.**
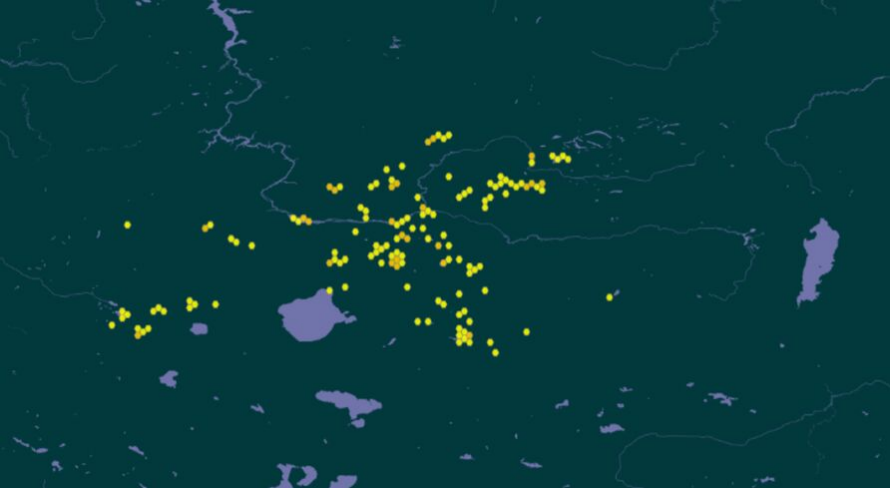
Location scheme (49°45' – 53°46' North latitude and 88°49' – 98°56' East longitude) of the studied waterbodies in the Republic of Tuva.

**Figure 2.**
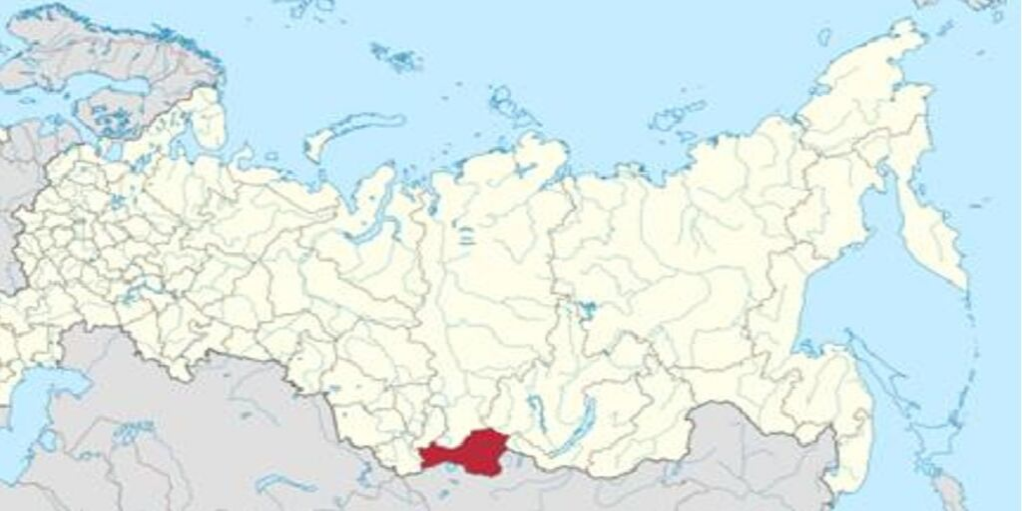
Layout of the studied waterbodies in the Republic of Tuva.

The studies encompassed the Republic of Tyva (Russian Federation), covering the basins of the Yenisey River, the Great Lakes Depression and the Selenga River (49°45' – 53°46' North latitude and 88°49' – 98°56' East longitude) (Figs 1, 2).

## Sampling methods

### Study extent

The presented dataset on the taxonomic composition and abundance of cladocerans in the Republic of Tyva is based on the authors' original samples. The species list includes only native species; no invasive species were recorded.

### Sampling description

**Figure 3.**
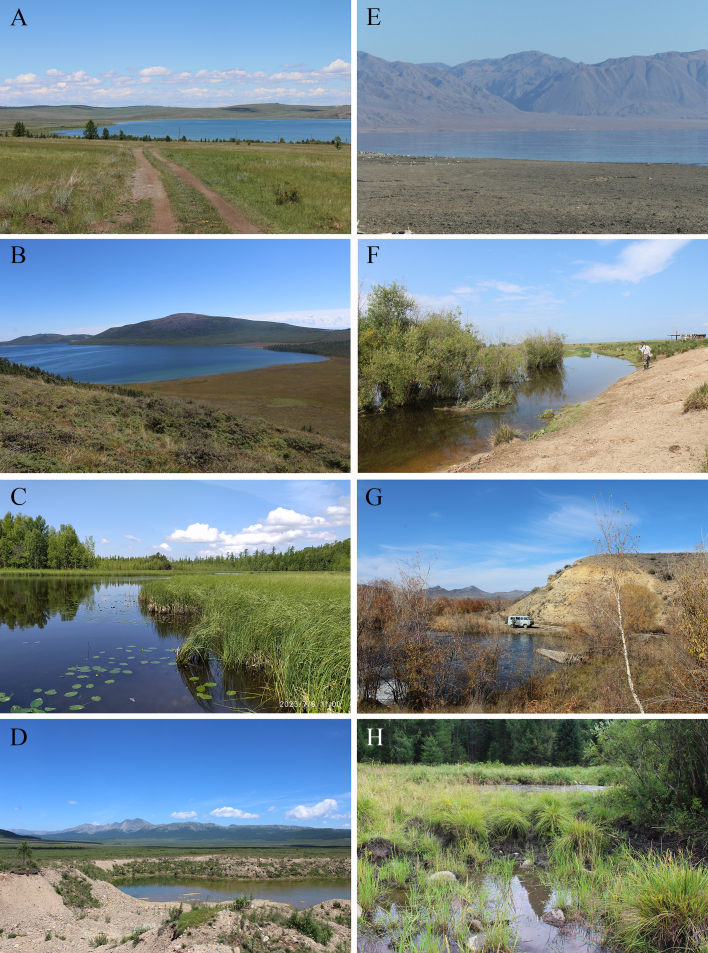
Waterbodies of the Republic of Tyva, Russian Federation. **A** Lake Chagytay, Ulug-Khem depression; **B** Lake Sut-Khol, Alash Plateau; **C** Lake Azas, Todzhin depression; **D** former sand quarry near River Ulug-O, Todzha; **E** Biy-Khem basin; Sayano-Shushenskoe Reservoir; **F** River Durgen, mid-stream, Ulug-Khem depression; **G** River Mezhegey, Ulug-Khem depression; **H** River Tapsa floodplain, Biy-Khem basin. Photos by N.A. Kirova.

All samples were collected using plankton nets from the shore and from a boat in the case of large lakes and reservoirs (Fig. 3). Species identification was carried out, based on the latest guides to cladocerans (Korovchinsky et al. 2021a).

Species lists were checked against GBIF Backbone Taxonomy (2021) (GBIF Secretariat 2023).

### Quality control

Species identification was primarily based on the latest monographs, developed with the participation of A.A. Kotov (Kotov et al. 2010, Korovchinsky et al. 2021a). Juvenile specimens were identified to the genus level and excluded from this dataset.

In the course of our previous studies on the cladocerans of Tyva, several papers were published in international journals (Kotov 1999, Kirova 2021, Sinev et al. 2021, Kirova and Oydup 2022), reporting several new records for Russia. The taxonomic nomenclature is given in accordance with the taxonomic checklist of GBIF Backbone Taxonomy (GBIF Secretariat 2023); scientific names were further validated using the GBIF species matching tool. To facilitate publication on the GBIF network, the records were formatted in accordance with Darwin Core specifications (Wieczorek et al. 2012).

### Step description

Zooplankton samples were collected by filtering water through a plankton net, either in the sublittoral zone or open water from a boat. The samples were concentrated to 100 ml and fixed in a 4% formalin solution according to conventional method (Korovchinsky et al. 2021a).

Zooplankton specimens were initially examined using a Leica MZ7.5 stereoscopic microscope (Leica Microsystems, Germany). Individual specimens were picked with a plastic pipette, placed in drops of a glycerol-water solution (1:1) and analysed using an Olympus CX41 light microscope (Olympus Corporation, Japan). After processing, all samples were stored in the first author’s personal collection for further taxonomic and morphological studies. Initially, all records were incorporated into the last author’s local database in Microsoft Access. They were then prepared for import to GBIF using Microsoft Excel. The dataset field names were chosen according to Darwin Core standards (Wieczorek et al. 2012).

We have also calculated the rate of:

1) benthic-phytophylous taxa (Suppl. material 1) living in the macroptype thickets in the littoral zone, on the bottom or in the upper layer of bottom sediments (it is difficult to distinguish truly phytophylous and bottom species in case of the cladocerans, as many taxa could live in both biotopes);

2) planktonic taxa living in the water column both in pelagic or sublittoral zone;

3) neustonic taxa spending much time attached to the bottom side of the water surface film.

Information on the mode of life was extracted from previously published data (Korovchinsky et al. 2021b).

**Figure 4.**
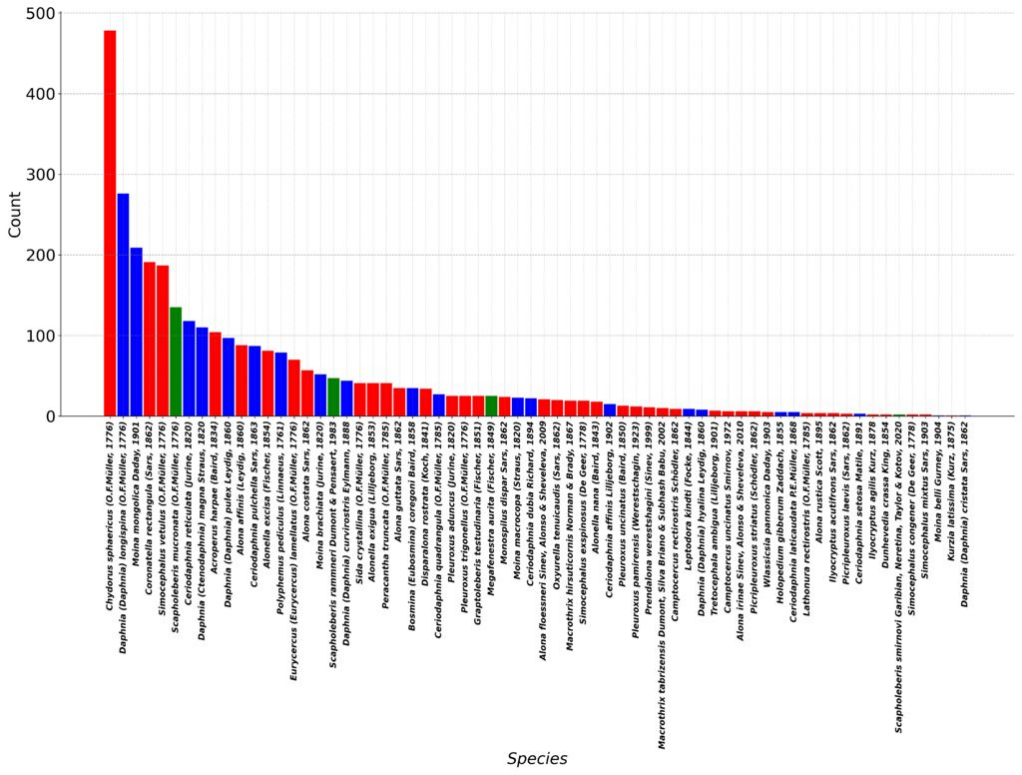
Numbers of each taxon records in 902 locations in the Republic of Tuva: benthic-phytophylous (red), planktonic (blue) and neustonic (green) taxa.

Unfortunately, to date, a zoogeographic analysis of our data seems to be premature as many revealed taxa represent, in reality, groups of non-revised congener species with presumably different geographic ranges and ecological preferences (Korovchinsky et al. 2021b). The rate of benthic-phytophylous/planktonic/neustonic taxa was 56.6/38.1/5.3 (in %) for the species and 60.0/38.4/6.6 for records; therefore, benthic-phytophylous taxa dominated in Tuva waterbodies (Fig. 4) similarly to other waterbodies of Eurasia (Kotov et al. 2013, Korovchinsky et al. 2021b. Eurybiotic benthic-phytophylous taxon Chydorus sphaericus was found in most waterbodies, while planktonic Daphnia longispina was very common in fresh waterbodies and *Moina mongolica* dominated in brackish waterbodies.

## Geographic coverage

### Description

The Republic of Tyva is located in the middle of the Asian continent, occupying a large territory (ca. 168,600 km^2^) in southern Siberia and bordering Mongolia in the south. Landscapes of the Republic are very diverse,including mountain ridges and plateaus (mountains occupy 82% of the territory) and intermountain river valleys (Sidorenko 1966). The main features of the landscape were formed there during the Caenozoic (Bazarov 1986). In general, Tyva is ringed by high mountains (the Sayan Mountains in the north and the East Siberian Mountains in the east), with the flat Tuva Basin located in its central and southern parts.

The climate is sharply continental. Winters are long, frosty and windless; springs are short, clear, windy and dry; summers are short, cool and dry in the mountains, but hot and dry in the lowlands; autumns are dry and sunny, with occasional periods of heat. Precipitation in the lowlands ranges from approximately 150 to 400 mm per year, while in mountainous areas, it varies from 400–600 mm to 800–1000 mm per year, with maximum precipitation occurring in summer. The frost-free period lasts 90–116 days and the growing season spans 150–160 days. Permafrost is common throughout the territory (Efimtsev 1957, Bakhtin 1968).

Diverse latitudes and climates create a variety of aquatic environments, ranging from high-altitude mountain lakes to "lowland" rivers and temporary waterbodies at approximately 1000 m a.s.l. Most of the Republic of Tyva lies within the Ulug Khem (Yenisey River) Basin, which is formed by the confluence of the Biy Khem (Large Yenisey) and Kaa Khem (Little Yenisey). A smaller portion of the territory belongs to the endorheic Mongolian Great Lakes Depression, while a minor area is part of the Selenga River Basin. Approximately 6,700 lakes are recorded within the Republic. The diverse landscape and climatic conditions contribute to the seasonal hydrological dynamics across different types of waterbodies (Klopova 1957, Arakchaa and Kurbatskaya 1998).

### Coordinates

 and 49°45' – 53°46' North latitude Latitude; and 88°49' – 98°56' East longitude Longitude.

## Taxonomic coverage

### Description

The dataset contains information on 76 species from 35 genera documented in the Republic of Tuva, with each record georeferenced. The following families are represented: Bosminidae, Cercopagididae, Chydoridae, Daphniidae, Eurycercidae, Holopedidae, Ilyocryptidae, Leptodoridae, Macrothricidae, Moinidae, Polyphemidae and Sididae. The dataset comprises a total of 3,599 records (Dadykin et al. 2024).

The umber of revealed species represents 27% of the total number of species in the whole of Northern Eurasia (76 vs. 287, see Korovchinsky et al. (2021a)) and 28% of the species in the Palaearctic (Kotov et al. 2013). This number is comparable with that for Tajikistan (75, see Dadykin et al. (2024)) which also includes mountain and lowland portions.

Fortunately, no non-indigenous taxa were identified. However, it should be noted that only a morphological approach was used and molecular methods could potentially detect such taxa within cryptic species complexes.

## Temporal coverage

### Notes

The presented dataset contains information on the diversity of cladocerans in the Republic of Tyva during the vegetation seasons of 1993 to 2022 in the Yenisey and Selenga River Basins and in the Depression of Great Mongolian Lakes.

## Collection data

### Collection name

The zooplankton collections of the Tuvinian Institute for the Exploration of Natural Resources of the Siberian Branch of Russian Academy of Sciences and the collection of the laboratory for aquatic ecology and invasions of A.N. Severtsov Institute of Ecology and Ecology of RAS.

## Usage licence

### Usage licence

Other

### IP rights notes

CC BY 4.0

## Data resources

### Data package title

Diversity of the cladocerans (Crustacea, Branchiopoda) in the Republic of Tyva, Russian Federation

### Resource link

https://doi.org/10.15468/ns642b

### Alternative identifiers

https://www.gbif.org/dataset/fca47d2f-0188-4c55-b46a-20aa05ed76c7

### Number of data sets

1

### Data set 1.

#### Data set name

Diversity of the cladocerans (Crustacea, Branchiopoda) in Republic of Tyva, Russian Federation

#### Data format

Darwin Core

#### Download URL

https://api.gbif.org/v1/occurrence/download/request/0080518-250525065834625.zip

#### Description

The dataset provides information on 76 species from 35 genera found in the Republic of Tuva, each documented with corresponding geographic coordinates (Kirova et al. 2024). These taxa belong to the following families: Bosminidae (2 species from 1 genus), Cercopagididae (1 species from 1 genus), Chydoridae (30 species from 17 genera), Daphniidae (26 species from 5 genera), Eurycercidae (1 species from 1 genus), Holopedidae (1 species from 1 genus), Ilyocryptidae (2 species from 1 genus), Leptodoridae (1 species from 1 genus), Macrothricidae (5 species from 3 genera), Moinidae (5 species from 1 genus), Polyphemidae (1 species from 1 genus) and Sididae (2 species from 2 genera). The dataset comprises 3,599 records.

Each observation in the dataset includes basic metadata: location (latitude and longitude), observation date, observer name and identifier. Coordinates were recorded in situ using a Garmin eTrex 32x (Garmin Ltd., USA).

**Data set 1.** 

Column label	Column description
type	The nature or genre of the resource.
eventID	An identifier for the set of information associated with an event (something that occurs at a place and time).
eventDate	The date when material from the trap was collected or the range of dates during which the trap collected material.
basisOfRecord	The specific nature of the data record.
occurrenceID	An identifier for the Occurrence (as opposed to a particular digital record of the occurrence).
continent	The name of the continent in which the location occurs.
country	Country of the sampling site.
countryCode	The standard code for the country in which the Location occurs.
stateProvince	The name of the next smaller administrative region than country (state, province, canton, department, region etc.) in which the location occurs.
higherGeography	The name of geographic sub-regions.
locationID	Identifier of sampling location for this dataset.
location	The name of the waterbody in which the location occurs.
samplingProtocol	The mode of sampling used.
locationRemarks	Remarks on the waterbody type and water salinity.
decimalLatitude	The geographic latitude of location in decimal degrees.
decimalLongitude	The geographic longitude of location in decimal degrees.
geodeticDatum	The ellipsoid, geodetic datum or spatial reference system (SRS), upon which the geographic coordinates given in decimalLatitude and decimalLongitude are based.
coordinateUncertaintyInMetres	The horizontal distance (in metres) from the given decimalLatitude and decimalLongitude describing the smallest circle containing the whole of the terms.
recordedBy	The full scientific name of the class in which the taxon is classified.
identifiedBy	A person, who assigned the Taxon to the subject.
kingdom	The full scientific name of the kingdom in which the taxon is classified.
phylum	The full scientific name of the phylum or division in which the taxon is classified.
class	The full scientific name of the class in which the taxon is classified.
order	The full scientific name of the order in which the taxon is classified.
family	The full scientific name of the family in which the taxon is classified.
genus	The full scientific name of the genus in which the taxon is classified.
taxonRemarks	The comments on alternative meaning of the species order.
taxonID	The identifier for the set of taxon information (data associated with the Taxon class). Specific identifier to the dataset.
scientificName	The name with authorship applied on the first identification of the specimen.
acceptedNameUsage	The specimen accepted name, with authorship.
specificEpithet	The name of the first or species epithet of the scientificName.
scientificNameAuthorship	The authorship information for the scientificName.
taxonRank	The taxonomic rank of the most specific name in the scientificName.
establishmentMeans	Statement about whether an organism has been introduced to a given place and time through the direct or indirect activity of modern humans.

## Supplementary Material

6D9C9C83-D06D-5D7B-9B52-D2D65AF6C844 10.3897/BDJ.13.e163656.suppl1 Supplementary material 1 Belonging of each cladoceran species to taxa Data type Supplementary Table Brief description Belonging of each cladoceran species found in the Republic of Tuva to benthic-phytophylous/planktonic/neustonic taxa. File: oo_1415433.xlsx https://binary.pensoft.net/file/1415433 Kirova N., Kirova V., Kotov A.
